# Cooperation of acylglycerol hydrolases in neuronal lipolysis

**DOI:** 10.1016/j.jlr.2023.100462

**Published:** 2023-10-21

**Authors:** Liqing Yu

**Affiliations:** Division of Endocrinology, Diabetes and Nutrition, Department of Medicine, University of Maryland School of Medicine, Baltimore, MD, USA

**Keywords:** lipid droplet, brain, neuron, hereditary spastic paraplegia, DDHD-domain containing 2, adipose triglyceride lipase, hormone sensitive lipase, intracellular phospholipase

## Abstract

Genetic and biochemical evidence has established DDHD-domain containing 2 (DDHD2) as the principal triacylglycerol (TAG) hydrolase in neuronal lipolysis of cytosolic lipid droplets. In this issue of *Journal of Lipid Research*, Hofer *et al.* report that DDHD2 cooperates with adipose triglyceride lipase, the principal TAG hydrolase in adipose lipolysis, contributing to cytosolic hydrolysis of both TAG and diacylglycerols in murine neuroblastoma cells and primary cortical neurons via different configurations of the lipases. This finding highlights the complexity of cytosolic acylglycerol hydrolysis and raises many new questions in the field of lipid metabolism.

Lipid droplet (LD) is an evolutionarily conserved organelle that is composed of a hydrophobic neutral lipid core and a phospholipid monolayer membrane surface (1, 2). In mammalian cells, the major neutral lipids in the core of LDs are triacylglycerols (TAGs) and cholesterol esters. There are many proteins that are constitutively associated with or recruited to the LD surface in a cell type–dependent manner. The LD association and dissociation of proteins are dynamically regulated under different pathophysiological conditions through various cell signaling pathways. It is generally accepted that LDs are formed from the endoplasmic reticulum (ER) (2, 3, 4). When biogenesis exceeds turnover, such as in the fed state, LDs accumulate in cells. During increased energy demand or prolonged starvation, LD turnover is stimulated, and the energy-rich TAG molecules are hydrolyzed via a lipolytic process that is catalyzed by specific lipases and regulatory factors. In adipocytes, the rate-limiting enzyme of cytosolic LD lipolysis is adipose triglyceride lipase (ATGL) that requires the coactivator comparative gene identification-58 CGI-58 (also known as α/β-hydrolase domain containing 5 or Abhd5) to achieve its maximum TAG hydrolytic activity (5). ATGL preferentially cleaves the fatty acyl chain at the *sn*-2 position from a TAG molecule, generating *sn*-1,3-diacylglycerol (*sn*-1,3-DAG). Diacylglycerol (DAG) is further hydrolyzed by hormone sensitive lipase (HSL), producing a monoacylglycerol (MAG) and a fatty acid. The last fatty acid in the MAG is cleaved by monoacylglycerol lipase. Through this lipolytic cascade, adipose tissue mobilizes its energy stores for utilization by other vital organs.

In the nervous system, LDs are observed more frequently in glia than neurons (6, 7, 8). Whereas ATGL has been well documented as the principal TAG hydrolase during the cytosolic LD lipolysis in many peripheral tissue cells (9) and perhaps in brain glial cells (8), its counterpart in neurons has just begun to emerge. In 2012, Schuurs-Hoeijmakers *et al.* reported that the complex hereditary spastic paraplegia (HSP) subtype SPG54, an autosomal recessive genetic disease, was caused by mutations in human DDHD-domain containing 2 (*DDHD2*) gene (10), which was subsequently confirmed by several other groups (11, 12, 13). Patients with SPG54 manifest early-onset (<2 years) spastic paraplegia, intellectual disability, thin corpus callosum, and a lipid peak under cerebral magnetic resonance spectroscopy. This lipid peak results from LD deposition in neurons throughout the brain. Although ATGL is expressed in the brain, including glial cells and certain neurons, mutations in *Atgl* gene do not cause widespread brain LD accumulation (14, 15). Recent animal and biochemical studies have established DDHD2 as the principal TAG hydrolase in neurons (16, 17).

DDHD2 belongs to the mammalian intracellular phospholipase A_1_ (iPLA_1_) family, a subgroup of serine hydrolases. This protein subfamily has three members, including DDHD1 (also annotated as iPLA_1_α or PA-PLA_1_), DDHD2 (also annotated as iPLA_1_γ or KIAA0725p), and Sec23ip (also known as p125 or iPLA_1_β). They share a conserved lipase motif (GxSxG) and a DDHD domain that was named after its three aspartates and one histidine residues. Nakajima *et al.* was the first to identify KIAA0725p as a phosphatidic acid (PA)-preferring PLA_1_ that also hydrolyzes phosphatidylserine (PS) and phosphatidylcholine (PC) but not phosphatidylethanolamine based on the in vitro substrate assays (18). The in vivo significance of this in vitro PLA_1_ activity has yet to be established because global knockout of *Ddhd2* in mice does not alter the contents of phospholipids in the brain (16). Moreover, Sato *et al.* observed that DDHD2 is associated with the Golgi/ER-Golgi intermediate compartment in a phosphoinositide-dependent manner in 293T cells and rapidly cycles between the Golgi and cytosol in HeLa cells (19, 20). They also observed that DDHD2 contributes to efficient membrane trafficking from the Golgi apparatus to the plasm membrane but not to the brefeldin A-induced Golgi-to-ER retrograde transport in HeLa cells (19). However, Inloes *et al.* showed that DDHD2 localizes to a perinuclear membrane structure that is adjacent to but does not overlap with markers of classical ER or cis-Golgi compartments in Cos-7 cells (17). In this issue of *Journal of Lipid Research* (21), Hofer *et al.* observed that endogenous DDHD2 protein is enriched in the perinuclear region and largely overlaps with the Golgi marker GM130 in Neuro-2a neuroblastoma cells. They further showed that endogenous DDHD2 in murine primary cortical neurons localizes to the vesicular and reticular structures that are similar to those stained by GM130. Clearly, the identity of the DDHD2-positive membrane structure has yet to be defined. Despite this, there is a consensus that DDHD2 does not localize to the LD surface.

Although genetic evidence and in vitro hydrolase activity assays have established DDHD2 as the principal TAG hydrolase in brain neurons, a few pieces of evidence also suggested a role of ATGL in neuronal lipolysis. For example, In *Caenorhabditis* elegans (*C*. elegans), a mutant of ATGL-1 (homolog of mammalian ATGL) or LID-1 (homolog of mammalian CGI-58, the coactivator of ATGL) accumulates LDs in neurons (22). In Drosophila, the ATGL homolog Brummer was shown to regulate neuronal lipolysis post starvation in males (23). Pharmacological inhibition of ATGL’s TAG hydrolase activity increases cytosolic LDs in neural stem/progenitor cells isolated from the mouse brain (24). Yang *et al.* showed that treatment with either the ATGL inhibitor Atglistatin or the DDHD2 inhibitor KLH45 induces LD accumulation in the dorsal root ganglion neurons isolated from mice (25). Additionally, they showed that the shRNA-mediated silencing of ATGL or DDHD2 in the eyes of mice blocks axon regeneration following optic nerve crush. Furthermore, they demonstrated that pharmacological inhibition of ATGL or DDHD2 impairs axon elongation of cultured dorsal root ganglion neurons and axon regeneration of the injured sciatic nerve in mice (25). Hundahl *et al.* reported that ATGL expression can be detected in certain neurons of the mouse brain (15). In this issue of *Journal of Lipid Research* (21), Hofer *et al.* showed that murine Neuro-2a neuroblastoma cells express both ATGL and DDHD2 proteins. Their pharmacological inhibition studies implied that murine primary cortical neurons also express both proteins. These observations raise several interesting questions. For instance, why do the two major cytosolic TAG hydrolases coexist in a cell? Do they cooperate in cytosolic LD lipolysis? If yes, how? Is this cooperation cell type-specific?

To address the aforementioned questions, Hofer *et al.* performed detailed biochemical, pharmacological, and cell-based studies (21). First, they measured in vitro hydrolase activities toward TAG, DAG, MAG, and PC of the cellular extract of Cos-7 cells transiently expressing an equal amount of His_6_-tagged recombinant DDHD2, DDHD1, Sec23ip, HSL, or ATGL protein. Consistent with previous reports (16, 17), DDHD2, like ATGL and HSL, showed high in vitro TAG hydrolase activity. The PC hydrolase activity was only observed in the cellular extract containing DDHD2 or DDHD1. Whereas ATGL exclusively hydrolyzed TAG, DDHD2, like HSL, can hydrolyze TAG, DAG, and MAG. Next, they measured the effect of small molecule lipase inhibitors on the in vitro lipase activities of the cellular extract of Neuro-2a cells that express endogenous ATGL, HSL, and DDHD2 proteins. The DDHD2 inhibitor KLH45 but not the ATGL inhibitor Atglistatin or the HSL inhibitor 76–0079 reduced cellular hydrolase activities toward TAG, DAG, and MAG. Silencing of the endogenous DDHD2 had similar effects. Surprisingly, combined treatment with DDHD2, ATGL, and HSL inhibitors further reduced in vitro TAG and DAG hydrolase activities, suggesting that cytosolic lipases cooperate in acylglycerol hydrolysis. Despite a dominant contribution of DDHD2 to the acylglycerol hydrolase activities in the cellular extract of Neuro-2a cells, pharmacological inhibition of DDHD2 by KLH45 for 24 h failed to increase cellular levels of LDs or TAG in these cells. Only ATGL inhibition by Atglistatin for 24 h resulted in such an increase. The lipid turnover studies using [^14^C]oleic acid coupled with the small molecular inhibitors or shRNAs also demonstrated that it was ATGL not DDHD2 inhibition that caused TAG accumulation and reduced TAG breakdown or lipolysis. These data demonstrated a dominant role of ATGL in TAG catabolism and LD turnover in living Neuro-2a cells (Fig. 1). It should be noted that inhibition of HSL did not alter cellular LD or TAG levels, though HSL protein is expressed in these cells.

**Fig. 1:**
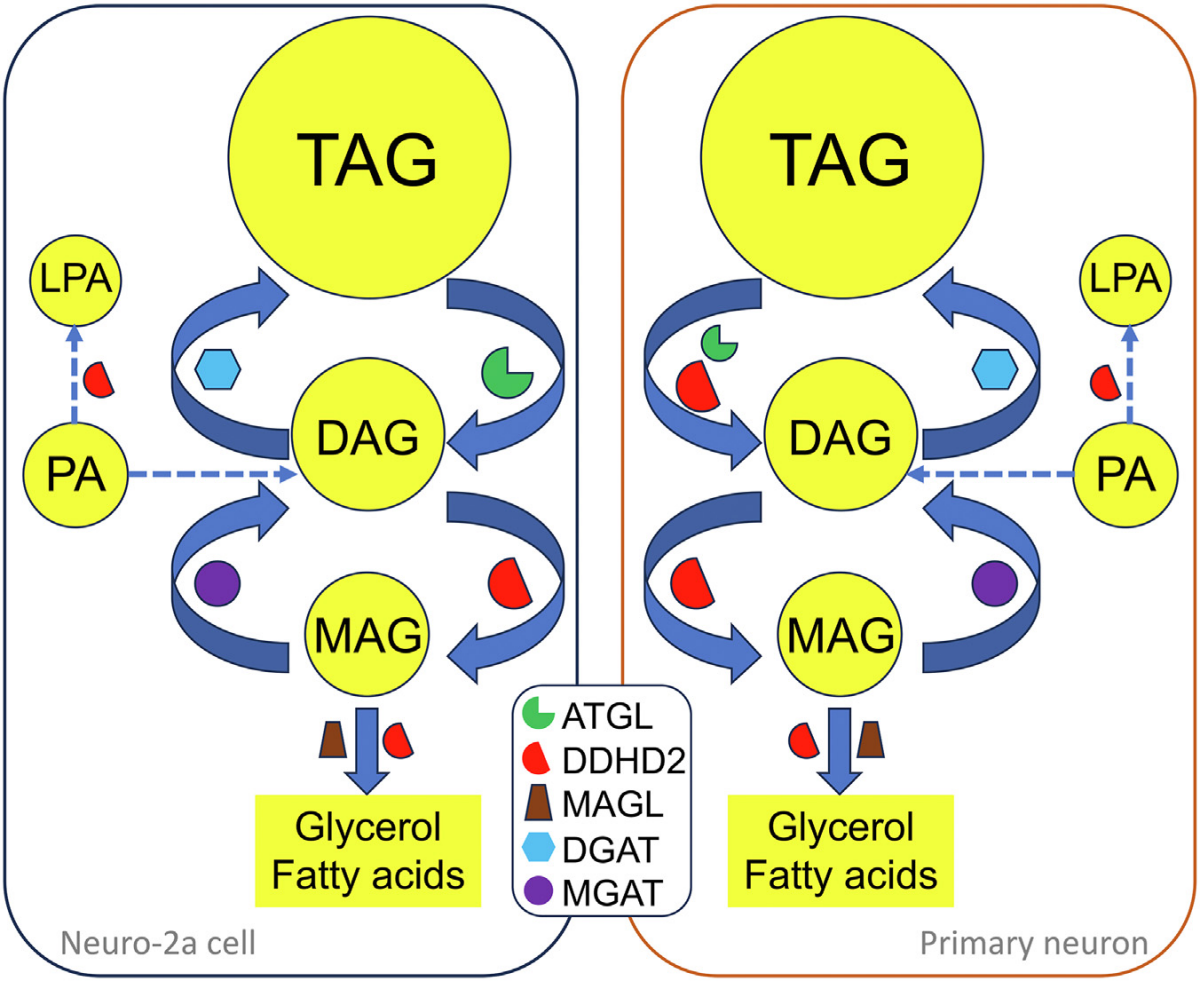
A proposed model for DDHD2 and ATGL cooperation in neuronal lipolysis. In Neuro-2a neuroblastoma cells, DDHD2 functions exclusively as a DAG hydrolase downstream of ATGL that is responsible for hydrolysis of TAG stored in cytosolic LDs. In murine primary cortical neurons, DDHD2 functions as a TAG/DAG dual hydrolase and cooperates with ATGL to achieve maximum TAG hydrolysis. When hydrolyzing acylglycerols at several steps, DDHD2 may limit TAG synthesis by reducing re-esterification of the lipolytic products. Hypothetically, if DDHD2 has phospholipase activity towards phosphatidic acid (PA) in vivo, it may limit cellular TAG by reducing DAG synthesis from PA. DGAT, diacylglycerol O-acyltransferase; LPA, lysophosphatidic acid; MAGL, monoacylglycerol lipase; MGAT, monoacylglycerol O-acyltransferase.

An important question is raised from Hofer’s findings in Neuro-2a cells. Why did the pharmacological inhibition of DDHD2, the major TAG hydrolase in the brain, have no effects on cellular levels of TAG and LDs in these neuroblastoma cells? Inloes *et al.* observed that a subchronic treatment was required for the DDHD2 inhibitor KLH45 to increase brain TAG in mice (15). Do these findings reflect a general property of KLH45 action in living cells or the time needed for KLH45 to reach and inhibit DDHD2? Will longer treatment of KLH45 increase levels of TAG and LDs in Neuro-2a cells? Does DDHD2, like HSL and ATGL, possess transacylase activity? Acute inhibition of transacylase activity might temporarily alleviate TAG accumulation. Do DDHD2 and ATGL have different sets (pools) of TAG substrates in living cells? Hofer *et al.* had some observations that suggested an interesting scenario (21). Considering the broad neutral lipid hydrolase activities of DDHD2, they tested whether DDHD2 predominantly affects cellular DAG catabolism in Neuro-2a cells by using DAG regiomers that are known to be derived from different metabolic pathways. *sn*-1,2-DAG is an intermediate of glycerolipid synthesis and glycerophospholipid breakdown by phospholipase C and phosphatidate phosphatase, whereas *sn*-1,3-DAG and *sn*-2,3-DAG are predominantly generated by the ATGL-mediated TAG hydrolysis (26, 27). To assess the effect of lipase inhibition on different DAG regiomers in Neuro-2a cells, they labeled cellular lipids with [^14^C]oleic acid and separated DAG regiomers by thin layer chromatography. Pharmacological inhibition or shRNA silencing of DDHD2 resulted in a significant increase in cellular *sn*-1,3-DAG. ATGL inhibition reduced the cellular *sn*-1,3-DAG, whereas HSL inhibition had no effect. Inhibition of each of these lipases did not alter cellular levels of the other DAG regiomers. Although DDHD2 has strong in vitro phospholipid hydrolase activity, its inhibition by KLH45 did not alter cellular levels of either PC, phosphatidylethanolamine, and PS, or their molecular species. These data demonstrated that DDHD2 is the principal DAG lipase downstream of ATGL in Neuro-2a cells (Fig. 1), resembling HSL in adipocytes. It was reported that *Hsl* knockout in mice caused a near absence of acylglycerol hydrolase activity in the brain extract (28) and that neuronal deletion of *Hsl* in hypothalamus can acutely reduce food intake in response to stress and high-fat diet feeding in mice (15). Here, Hofer *et al.* showed that HSL is not responsible for the hydrolysis of ATGL-derived DAGs in Neuro-2a cells (21). These findings together highlight the necessity of identifying the exact lipid substrate of each lipase in each cell type. Filling this knowledge gap would enable the cell type–specific control of neutral lipid lipolysis. It cannot be assumed that HSL is responsible for the hydrolysis of ATGL-generated DAG in all cell types, even when both lipases coexist.

In murine primary cortical neurons, DDHD2 and ATGL appear to adopt a different configuration. Hofer *et al.* observed that DDHD2 inhibition by KLH45 increased not only *sn*-1,3-DAG (1.7-fold) but also TAG (3-fold), suggesting that DDHD2 has TAG/DAG dual hydrolase activities in these neurons (21) (Fig. 1). Whereas HSL did not alter cellular TAG and *sn*-1,3-DAG levels, ATGL inhibition by Atglistatin increased cellular TAG 2.5-fold despite no effect on the murine brain’s in vitro TAG hydrolase activity. Interestingly, combined inhibition of DDHD2 and ATGL further increased cellular TAG (3.9-fold). The authors did not report the effect of DDHD2 inhibition on cellular phospholipids or ATGL inhibition on cellular *sn*-1,3-DAG. Nonetheless, the data collectively demonstrated that DDHD2 and ATGL both participate in cellular TAG hydrolysis in murine primary cortical neurons (Fig. 1).

Like many novel findings, those by Hofer *et al.* raise more questions than answers. For example, what is the in vivo significance of their in vitro findings? If assuming that ATGL has similar functions in the brain, we would have to address several obvious questions. Why is the endogenous ATGL unable to substitute for DDHD2 to prevent neuronal TAG accumulation in patients with SPG45 or mice deficient in DDHD2? Why did ATGL deficiency not cause widespread LD accumulation in the brain while its inhibition caused LD accumulation in cultured neurons? Do glial cells express DDHD2 in addition to ATGL? If yes, was it DDHD2 that protected ATGL-deficient brain against both neuronal and glial LD accumulation? Hofer *et al.* speculated that “the contribution of ATGL to neuronal TAG catabolism in vivo might be restricted to specific development stages, pathophysiological conditions, or cell populations.” While this speculation is laudable, additional experiments are clearly needed to provide direct in vivo evidence. One of such experiments could be genetic deletion of ATGL and/or DDHD2 in specific neurons in a temporal fashion. CGI-58, the coactivator of ATGL, was found to be associated with the LDs accumulated in the brain of *Ddhd2* knockout mice (17). Given the low expression of ATGL in brain neurons, it might be worth testing whether neuronal overexpression of ATGL can prevent LD accumulation in *Ddhd2* knockout mice. Additionally, efforts could be made to detailed mapping of lipase expression and subcellular locations in different neurons and glial cells of normal brains in comparison to LD-containing brains such as those caused by aging (29) and neuronal degenerative diseases (6).

DDHD2 is expressed in many peripheral tissues with the highest expression in the brown and white adipose tissues (21). Despite this, DDHD2 is unable to substitute for ATGL or ATGL coactivator CGI-58 to hydrolyze TAG because adipose *Atgl* or *Cgi-58* knockout mice accumulate large LDs in the brown adipose tissue (30, 31, 32). These observations imply that DDHD2 and ATGL may not cooperate in TAG hydrolysis in peripheral tissues. Hofer *et al.* showed that the DDHD2 inhibitor KLH45 can only inhibit the in vitro TAG hydrolase activity in the homogenate of murine brain but other tissues (21). Does KLH45 engage a neural factor to inhibit DDHD2’s TAG hydrolase activity? Is a coactivator required for the activation of DDHD2’s acylglycerol hydrolase activities? If yes, is this coactivator exclusively expressed in the brain? It is time to catalog DDHD2-interacting proteins in different cell types (e.g., neurons and adipocytes) and study whether these proteins regulate DDHD2’s various enzymatic activities.

DDHD2 is not a LD-associated protein. How does DDHD2 then gain access to its neutral lipid substrates that are often enclosed in the core of cytosolic LDs? It was speculated that DDHD2 may do so via organelle contact sites or transient translocation to LDs from the cytosol. DDHD2 is enriched in the perinuclear membrane structure in many cell types. Is this structure a special subpopulation of ER and cis-Golgi membranes that are involved in the biosynthesis of special lipids? DDHD2 has strong in vitro phospholipase activity towards PA (18), and PA is a precursor of DAG and TAG synthesis. Hypothetically, if DDHD2 can function as a PA phospholipase in vivo in this perinuclear membrane structure, its deficiency may promote TAG accumulation by raising PA and associated DAG/TAG synthesis (Fig. 1). Additionally, if DDHD2 can function as a DAG hydrolase in these membranes, its deficiency may elevate membrane DAG, leading to an increase in cellular TAG via DAG esterification (Fig. 1). Furthermore, DDHD2 was shown to efficiently regulate membrane trafficking from the Golgi apparatus to the plasma membrane (19). If this function of DDHD2 is involved in neuronal-to-glial lipid transport, deficiency of DDHD2 may cause neuronal lipid accumulation.

The discovery of *DDHD2* mutations as the cause of a complex HSP with widespread LD accumulation in the brain demonstrates a key role of neuronal LD formation and turnover in the regulation of central nervous system function. We are just beginning to glimpse DDHD2’s function and how DDHD2 integrates with other lipases to regulate acylglycerol and perhaps phospholipid hydrolysis. To molecularly define how DDHD2 governs cellular lipid homeostasis, the identity of its subcellular locations and the proteins it interacts with must be established in a cell type-specific manner and under specific pathophysiological conditions. It remains completely unknown whether DDHD2 regulates acylglycerol/phospholipid synthesis and cell signaling. Molecular elucidation of DDHD2’s role in central nervous system pathophysiology may shed light on the therapeutic targets for the treatment of HSP and other neurological disease.

## Conflict of interest

The author declares that he has no conflict of interest with the contents of this article.

## References

[1] Tauchi-Sato K., Ozeki S., Houjou T., Taguchi R., Fujimoto T. (2002). The surface of lipid droplets is a phospholipid monolayer with a unique fatty Acid composition. J. Biol. Chem..

[2] Brasaemle D.L., Wolins N.E. (2012). Packaging of fat: an evolving model of lipid droplet assembly and expansion. J. Biol. Chem..

[3] Walther T.C., Chung J., Farese R.V. (2017). Lipid droplet biogenesis. Annu. Rev. Cell Dev. Biol..

[4] Zadoorian A., Du X., Yang H. (2023). Lipid droplet biogenesis and functions in health and disease. Nat. Rev. Endocrinol..

[5] Lass A., Zimmermann R., Haemmerle G., Riederer M., Schoiswohl G., Schweiger M. (2006). Adipose triglyceride lipase-mediated lipolysis of cellular fat stores is activated by CGI-58 and defective in Chanarin-Dorfman Syndrome. Cell Metab..

[6] Ralhan I., Chang C.L., Lippincott-Schwartz J., Ioannou M.S. (2021). Lipid droplets in the nervous system. J. Cell Biol..

[7] Pennetta G., Welte M.A. (2018). Emerging links between lipid droplets and motor neuron diseases. Dev. Cell.

[8] Islimye E., Girard V., Gould A.P. (2022). Functions of stress-induced lipid droplets in the nervous system. Front. Cell Dev. Biol..

[9] Grabner G.F., Xie H., Schweiger M., Zechner R. (2021). Lipolysis: cellular mechanisms for lipid mobilization from fat stores. Nat. Metab..

[10] Schuurs-Hoeijmakers J.H., Geraghty M.T., Kamsteeg E.J., Ben-Salem S., de Bot S.T., Nijhof B. (2012). Mutations in DDHD2, encoding an intracellular phospholipase A(1), cause a recessive form of complex hereditary spastic paraplegia. Am. J. Hum. Genet..

[11] Gonzalez M., Nampoothiri S., Kornblum C., Oteyza A.C., Walter J., Konidari I. (2013). Mutations in phospholipase DDHD2 cause autosomal recessive hereditary spastic paraplegia (SPG54). Eur. J. Hum. Genet..

[12] Citterio A., Arnoldi A., Panzeri E., D'Angelo M.G., Filosto M., Dilena R. (2014). Mutations in CYP2U1, DDHD2 and GBA2 genes are rare causes of complicated forms of hereditary spastic paraparesis. J. Neurol..

[13] Chou Y.T., Hsu S.L., Tsai Y.S., Lu Y.J., Yu K.W., Wu H.M. (2023). Biallelic DDHD2 mutations in patients with adult-onset complex hereditary spastic paraplegia. Ann. Clin. Transl. Neurol..

[14] Etschmaier K., Becker T., Eichmann T.O., Schweinzer C., Scholler M., Tam-Amersdorfer C. (2011). Adipose triglyceride lipase affects triacylglycerol metabolism at brain barriers. J. Neurochem..

[15] Hundahl C., Kotzbeck P., Burm H.B., Christiansen S.H., Torz L., Helge A.W. (2021). Hypothalamic hormone-sensitive lipase regulates appetite and energy homeostasis. Mol. Metab..

[16] Inloes J.M., Hsu K.L., Dix M.M., Viader A., Masuda K., Takei T. (2014). The hereditary spastic paraplegia-related enzyme DDHD2 is a principal brain triglyceride lipase. Proc. Natl. Acad. Sci. U. S. A..

[17] Inloes J.M., Kiosses W.B., Wang H., Walther T.C., Farese R.V., Cravatt B.F. (2018). Functional contribution of the spastic paraplegia-related triglyceride hydrolase DDHD2 to the formation and content of lipid droplets. Biochemistry.

[18] Nakajima K., Sonoda H., Mizoguchi T., Aoki J., Arai H., Nagahama M. (2002). A novel phospholipase A1 with sequence homology to a mammalian Sec23p-interacting protein, p125. J. Biol. Chem..

[19] Sato S., Inoue H., Kogure T., Tagaya M., Tani K. (2010). Golgi-localized KIAA0725p regulates membrane trafficking from the Golgi apparatus to the plasma membrane in mammalian cells. FEBS Lett..

[20] Inoue H., Baba T., Sato S., Ohtsuki R., Takemori A., Watanabe T. (2012). Roles of SAM and DDHD domains in mammalian intracellular phospholipase A1 KIAA0725p. Biochim. Biophys. Acta.

[21] Hofer P., Grabner G.F., König M., Xie H., Bulfon D., Ludwig A.E. (2023). Cooperative lipolytic control of neuronal triacylglycerol by spastic paraplegia-associated enzyme DDHD2 and ATGL. J. Lipid Res..

[22] Yang L., Liang J., Lam S.M., Yavuz A., Shui G., Ding M. (2020). Neuronal lipolysis participates in PUFA-mediated neural function and neurodegeneration. EMBO Rep..

[23] Wat L.W., Chao C., Bartlett R., Buchanan J.L., Millington J.W., Chih H.J. (2020). A role for triglyceride lipase brummer in the regulation of sex differences in Drosophila fat storage and breakdown. PLoS Biol..

[24] Ramosaj M., Madsen S., Maillard V., Scandella V., Sudria-Lopez D., Yuizumi N. (2021). Lipid droplet availability affects neural stem/progenitor cell metabolism and proliferation. Nat. Commun..

[25] Yang C., Wang X., Wang J., Wang X., Chen W., Lu N. (2020). Rewiring neuronal glycerolipid metabolism determines the extent of axon regeneration. Neuron.

[26] Eichmann T.O., Kumari M., Haas J.T., Farese R.V., Zimmermann R., Lass A. (2012). Studies on the substrate and stereo/regioselectivity of adipose triglyceride lipase, hormone-sensitive lipase, and diacylglycerol-O-acyltransferases. J. Biol. Chem..

[27] Eichmann T.O., Lass A. (2015). DAG tales: the multiple faces of diacylglycerol--stereochemistry, metabolism, and signaling. Cell Mol. Life Sci..

[28] Haemmerle G., Zimmermann R., Hayn M., Theussl C., Waeg G., Wagner E. (2002). Hormone-sensitive lipase deficiency in mice causes diglyceride accumulation in adipose tissue, muscle, and testis. J. Biol. Chem..

[29] Shimabukuro M.K., Langhi L.G., Cordeiro I., Brito J.M., Batista C.M., Mattson M.P. (2016). Lipid-laden cells differentially distributed in the aging brain are functionally active and correspond to distinct phenotypes. Sci. Rep..

[30] Schoiswohl G., Stefanovic-Racic M., Menke M.N., Wills R.C., Surlow B.A., Basantani M.K. (2015). Impact of reduced ATGL-mediated adipocyte lipolysis on obesity-associated insulin resistance and inflammation in male mice. Endocrinology.

[31] Shin H., Ma Y., Chanturiya T., Cao Q., Wang Y., Kadegowda A.K.G. (2017). Lipolysis in Brown adipocytes is not essential for cold-induced thermogenesis in mice. Cell Metab..

[32] Schreiber R., Diwoky C., Schoiswohl G., Feiler U., Wongsiriroj N., Abdellatif M. (2017). Cold-induced thermogenesis depends on ATGL-mediated lipolysis in cardiac muscle, but not Brown adipose tissue. Cell Metab..

